# Study protocol for the Health Outcomes in Pregnancy and Early Childhood (HOPE) Study: A mother-infant study in American Samoa

**DOI:** 10.1371/journal.pone.0326644

**Published:** 2025-09-15

**Authors:** Lacey W. Heinsberg, Miracle Loia, Susie Tasele, Kima Faasalele-Savusa, Jenna C. Carlson, Scott Anesi, Katie Desobry, Efren Yuchongco, Brian Fortuno Guevara, Aigaeiva Sesaga, Alice Iloilo, Va’atausili Tofaeono, Kaylynn Bryan, Tina Tauasosi-Posiulai, Erin E. Kershaw, Yvette P. Conley, Daniel E. Weeks, Nicola L. Hawley, Bethel T. Muasau-Howard

**Affiliations:** 1 Department of Health Promotion and Development, School of Nursing, University of Pittsburgh, Pittsburgh, Pennsylvania, United States of America; 2 Department of Human Genetics, School of Public Health, University of Pittsburgh, Pittsburgh, Pennsylvania, United States of America; 3 Obesity, Lifestyle, and Genetic Adaptations (OLaGA) Study Group; 4 Department of Biostatistics and Health Data Science, School of Public Health, University of Pittsburgh, Pittsburgh, Pennsylvania, United States of America; 5 Lyndon B Johnson Tropical Medical Center, Faga’alu, American Samoa; 6 American Samoa Community Cancer Coalition, Nu’uuli, American Samoa; 7 Department of Indigenous Health, School of Medicine and Health Sciences, University of North Dakota, Grand Forks, North Dakota, United States of America; 8 Department of Tropical Medicine, John A. Burns School of Medicine, University of Hawaii at Manoa, Honolulu, Hawaii, United States of America; 9 Division of Endocrinology and Metabolism, Department of Medicine, University of Pittsburgh, Pittsburgh, Pennsylvania, United States of America; 10 Department of Chronic Disease Epidemiology, Yale University School of Public Health, New Haven, Connecticut, United States of America; University of Tennessee Knoxville, UNITED STATES OF AMERICA

## Abstract

Pacific Islanders, including those in American Samoa, face a disproportionately high burden of gestational diabetes mellitus (GDM) and related sequalae of metabolic conditions. The *CREBRF* rs373863828 genetic variant, which is uniquely common among Pacific Islanders, has been paradoxically associated with higher body mass index (BMI) but lower risk of type 2 diabetes. While emerging evidence suggests this variant may influence both maternal metabolic outcomes and infant growth, studies in pregnancy and early life remain limited. The purpose of this paper is to describe the protocol for a study designed to address these gaps. The Health Outcomes in Pregnancy and Early Childhood (HOPE) Study is an observational, longitudinal cohort study that will enroll up to 180 Samoan pregnant women and their infants (target n = 150 dyads completing study protocols) in American Samoa, with follow-up through six months postpartum/postnatal. The study includes questionnaires, anthropometric measurements, and biospecimen collection. Genetic and epigenetic analyses will examine associations between maternal and infant *CREBRF* rs373863828 genotype, gestational diabetes status, infant body size, and cord blood DNA methylation. The study is approved by the Institutional Review Boards at the University of Pittsburgh, Yale University, and the American Samoa Department of Health, as well as the Lyndon B. Johnson Tropical Medical Center (American Samoa) Research Oversight Committee. Findings will be disseminated through peer-reviewed publications, conference presentations, and community reports.

## Introduction

Pacific Islander women in the United States (U.S.) face a disproportionately higher risk of gestational diabetes mellitus (GDM) compared to women of European ancestry (9.9–14.8% versus 2–6%, respectively) [1]. In American Samoa, GDM statistics far exceed these numbers, affecting up to 40% of Samoan women [2]. GDM not only impacts maternal health but also birth outcomes and long-term health of the offspring [3,4]. For example, children born to mothers with GDM have an increased risk of metabolic irregularities that can promote the intergenerational transmission of obesity and chronic disease [5,6].

The genetic marker *CREBRF* rs373863828 is receiving increasing attention for its unique health effects in Pacific Islander populations [7–9] and its potential to improve our understanding of GDM and related child health outcomes. The variant’s minor allele (A) is uniquely common among Pacific Islanders (minor allele frequency ~0.26) and is associated with *greater* body mass index (BMI) but paradoxically *lower* odds of type 2 diabetes among adults [8]. These associations have been replicated across multiple Pacific Islander populations [9–13], and early evidence suggests the variant may also protect against GDM [14].

Although research on early life, a critical window for long-term metabolic programming, is still limited, evidence suggests that *CREBRF* may also influence infant growth and body composition. In a sample of young children from Samoa recruited at birth and followed through 2 years, the presence of the A allele (AA/AG genotypes) was associated with greater bone and lean mass in infants [15] and greater height in toddlers [16] compared to those without the A allele (GG genotype), which may begin to explain its longer-term metabolic health benefits [17–19]. Additional findings indicate that the association with height persists into early childhood and that, by around age four, associations with more clinically visible obesity-related phenotypes, such as greater weight and abdominal circumference, also begin to emerge [20].

Despite these findings, most *CREBRF* research has focused either on maternal genetic contributions to GDM or offspring genetic influences on growth, without considering the complex interplay between maternal-offspring genetics. This is an important gap because pregnancy is a time of dynamic biological adaptation during which the maternal metabolic system shifts to prioritize nutrient delivery for fetal development [21]. This shift is thought to be shaped by the mother’s metabolic status and genetic makeup alongside hormonal factors produced by the placenta, which generally shares the same genetic profile as the fetus [22]. This suggests that GDM may not emerge in isolation, but instead from suboptimal maternal-fetal resource allocation influenced by both maternal and fetal genetics [22,23]. Fetal growth is similarly shaped by this dual influence—indirectly by maternal genetics, which govern the intrauterine environment [24], and directly by fetal genetics, which affect insulin and hormone production both before and after birth [25]. In the case of *CREBRF*, we hypothesize that maternal-fetal co-occurrence of the A allele synergistically provides greater protection against GDM compared to maternal genetics alone, leading to improved anthropometric outcomes in children via fetal programming. Supporting this broader framework, other studies have shown that maternal-offspring genotype combinations (e.g., at *IRS1* rs1801278 [26]) can modulate GDM risk and, in turn, infant outcomes.

Beyond genetic contributions, epigenetic mechanisms such as DNA methylation may also play a key role in shaping infant health. As a critical regulator of gene expression and a central mechanism in fetal metabolic programming, DNA methylation is influenced by both genetic and environmental factors during fetal development [27,28]. Given its importance in human health, it is essential to understand how maternal-infant *CREBRF* genotype combinations affect DNA methylation, and how these genetic effects may interact with metabolic exposures, such as GDM, to shape the infant epigenome. Likewise, and more broadly, it is also important to understand how DNA methylation at birth can impact infant growth and development as this may help identify biological pathways through which maternal-fetal genetic interactions and prenatal exposures influence long-term metabolic health.

To address these gaps, we have designed the Health Outcomes in Pregnancy and Early Childhood (HOPE) study, which aims to understand the health and wellness of American Samoan women and their children, with a specific focus on GDM and infant growth. The primary objective is to examine how maternal-infant *CREBRF* rs373863828 genotypes jointly contribute to GDM status, infant body size, and genome-wide DNA methylation in cord blood at birth. Secondary objectives include integrating social, environmental, and behavioral data to understand how these contextual factors shape health outcomes, as well as exploring additional contributors to maternal and child health in this population, including genetic (e.g., *BTNL9* rs200884524, another Pacific Islander-specific variant linked to cardiometabolic health [29]), genomic (e.g., microbiome composition, which may indirectly influence infant growth and immune development [30]), and environmental (e.g., per and polyfluoroalkyl substance concentrations, which have been linked to metabolic disruption [31]).

## Materials and methods

### Study design and overview

This ongoing, longitudinal, observational study (recruitment started April 2025) aims to enroll up to n = 180 Samoan pregnant women and their index offspring from American Samoa, with a target of n = 150 mother-infant dyads completing study protocols. Eligible women will be enrolled during their third trimester of pregnancy, and each dyad will be followed through the infant’s first six months of life. Participants will complete five study visits: one during pregnancy, one shortly after birth, and follow-up visits at 2, 4, and 6 months postpartum. As part of the study, mothers will complete questionnaires about themselves, their families, and their infants; physical measurements and biospecimens will be collected from both mothers and infants; and additional health data will be abstracted from medical records to supplement study assessments. Further details on study procedures are provided below.

### Ethics

The HOPE study has received Institutional Review Board (IRB) approval from both the University of Pittsburgh and Yale University, with the University of Pittsburgh serving as the IRB of record through a multi-site IRB agreement (STUDY24020055). Local and territorial approval has been granted by the American Samoa IRB. In addition, the study has been approved by the Lyndon B Johnson Tropical Medical Center (LBJ) Research Oversight Committee for all hospital-related activities.

### Setting

American Samoa is an unincorporated territory of the U.S. located approximately halfway between Hawaii and New Zealand in the South Pacific Ocean [32]. It consists of several islands, the largest and most populated being Tutuila, which is home to >90% of residents [32]. Tutuila measures roughly 21 miles in length and 3 miles across at the widest points.

The American Samoan population consists predominantly of Samoan individuals. Given its mountainous terrain, American Samoa is quite urbanized with the main population center being Tafuna, home to ~8,000 residents [33]. According to the U.S. Census Bureau, the 2020 population of American Samoa was 49,710, a decline from 55,519 in 2010 [34], reflecting significant out migration, largely to the mainland U.S., and a decline in birth rates. American Samoa was recently classified as a high-income economy by the World Bank (transition from upper-middle economy the previous year due to revision in population estimates based on the 2020 census [35]), although the gross national income of $18,017 per capita in 2022 [36] is far lower than the $78,035 in the U.S. as a whole [37].

American Samoa’s healthcare system, designated as medically underserved by the U.S. Health Resources and Services Administration (HRSA) [38], includes one tertiary care facility, the Lyndon B Johnson Tropical Medical Center (LBJ), and four federally qualified community health centers (FQHCs) operated by the Department of Health. Residents are automatically enrolled in Medicaid through a waiver under the Social Security Act, bypassing individual eligibility assessments [39]. All prenatal care in the territory is centralized through five locations: the four FQHCs as well as the obstetrics/gynecology clinic at LBJ Hospital. While prenatal care is distributed across these sites, LBJ Hospital is the only delivery facility on the island, with over 98% of births occurring there annually.

The health landscape in American Samoa has been shaped by complex historical dynamics, including foreign influence and multifaceted internal political, economic, and social systems. For example, as U.S. nationals without full political representation—and therefore no direct voice in federal decisions—American Samoans face structural barriers that affect healthcare access and broader resource allocation. Historical shifts in food systems, coupled with limited local infrastructure and geographic isolation, have further contributed to persistent health challenges.

Despite these challenges, the Samoan cultural values of *fa’aSamoa*—the Samoan way— emphasizes respect, reciprocity, and family and community ties [40], offering a powerful foundation for the future. This study, in partnership with the American Samoan community, incorporates Samoan frameworks such as *Teu le* va (maintaining respectful relationships [41]) and *Talanoa* (“talk story”, a dialogic approach rooted in storytelling [42,43]). By grounding the research in these values, we aim to ensure that knowledge sharing between researchers and the Samoan community occurs in a respectful and culturally safe environment, and that the resulting solutions to improve health outcomes for women and children are aligned with Samoan norms.

### Participants

HOPE study participants will be recruited from FQHC and LBJ prenatal care clinics and through flyers, social media advertising, and clinician referrals. Inclusion criteria for dyads include: (1) maternal age ≥ 18 years; (2) maternal report that the child has four Samoan grandparents (an empirically validated method of assessing ancestry in this setting [8], included due to the genetic focus of this study); (3) pregnancy gestation at or beyond 35 weeks (to limit heterogeneity related to extremely preterm birth); (4) singleton pregnancy (to avoid heterogeneity in growth patterns seen in multiples); (5) maternal plans to give birth at LBJ and reside in American Samoa for at least six months post-birth (to enable follow-up); (6) maternal completion of a standard-of-care 2-hour, 75g oral glucose tolerance test for GDM screening between 24 and 28 weeks’ gestation (to have a current best practice measure of GDM status); and (7) maternal intent to provide cord blood and saliva DNA specimens (to conserve limited study resources for the primary study goal, though participants are reminded at each visit that their involvement in each specific assessment is optional).

Exclusion criteria for dyads include: (a) pre-pregnancy maternal diabetes, defined by either self-report or a fasting glucose level of ≥126 mg/dL or HbA1c ≥ 6.5% during a standard prenatal visit before 12 weeks’ gestation; (b) maternal medical history of conditions that may interfere with maternal weight gain or fetal growth, such as prior bariatric surgery or congenital anomalies; or (c) insulin use as a first-line treatment for GDM (to reduce treatment-related heterogeneity).

For those eligible and interested, an orientation visit will be conducted by bilingual (English and Samoan) research assistants. Participant understanding will be assessed, and informed written consent will be obtained from the mother that covers her participation and that of her unborn child. Participants will receive a small baby gift at the time of their infant’s birth and monetary compensation for each completed visit that are aligned with the local cost of living and vary based on visit length.

### Study procedures

The HOPE study consists of five study visits: one before birth (Visit 1), one after birth (Visit 2), and follow-ups at 2, 4, and 6 months (Visits 3–5). Visits are estimated to range from 20–90 minutes based on visit-specific research activities outlined in Table 1. Whenever possible, visits will be conducted in person at our research center, the participant’s home, or during standard clinical appointments (based on participant preferences). When in-person visits are not feasible, questionnaire data will be collected by phone. All data will be collected by trained Samoan research staff from the Obesity, Lifestyle and Genetic Adaptations (OLaGA; www.olaga.org) research center in American Samoa, who are bilingual in Samoan and English. Many have prior healthcare training or experience, and all receive training in research ethics and study procedures before engaging in data collection.

**Table 1:** Study timeline and research activities overview.

a. Maternal data collection						
	Visit 1 (Before birth)	Infant birth	Visit 2 (After birth)	Visit 3 (2mo)	Visit 4 (4mo)	Visit 5 (6mo)
Saliva sampling	X					X
Capillary blood sampling	X					X
Physical measurements (height, weight, heart rate, blood pressure, and point-of-care HbA1c)	X			X	X	X
Body composition measurements				X	X	X
**b. **Infant data collection						
	**Visit 1 (Before birth)**	**Infant birth**	**Visit 2 (After birth)**	**Visit 3** **(2mo)**	**Visit 4** **(4mo)**	**Visit 5** **(6mo)**
Umbilical cord blood sampling		X				
Saliva sampling			X			X
Capillary blood sampling			X	X		X
Stool sampling			X	X		X
Physical measurements (weight, length, head/abdominal circumference, skinfold thicknesses)			X	X	X	X
**c. **Dyad and family data collection						
	**Visit 1 (Before birth)**	**Infant birth**	**Visit 2 (After birth)**	**Visit 3** **(2mo)**	**Visit 4** **(4mo)**	**Visit 5** **(6mo)**
Water sampling (tap water, from the home)				X		
Questionnaire administration	X		X	X	X	X

The ‘X’ symbol indicates that activity-specific data collection will occur for a given visit. Visit 1: Will occur between 35 weeks gestation and the onset of labor; Visit 2: Will occur within 0–14 days after birth; Visits 3–5: Will occur at approximately 2, 4, and 6 months of infant age, with a ± 4-week window around each target month. Note: Biospecimen collection not scheduled to occur at the 4 month visit to reduce participant burden.

#### Physical (anthropometric and clinical) measurements.

At Visits 1, 3, 4, and 5, research assistants will collect maternal height, weight, heart rate, blood pressure, and HbA1c measurements. Maternal height will be measured using a portable stadiometer (SECA, Hamburg, Germany), and weight will be recorded with a digital scale (Tanita Corporation of America, IL, USA) with participants wearing lightweight clothing. Anthropometric measurements will be taken in duplicate and averaged for analyses. Blood pressure and heart rate will be measured after a 10-minute seated rest period, with a 3-minute rest between readings, using an automated blood pressure monitor (Omron Healthcare). Three measurements will be taken and the last two will be averaged for analyses. A point-of-care device (PTS Diagnostics A1cNow + ™ Systems) will be used to measure HbA1c via capillary blood collected from a finger stick after the finger is cleaned with an alcohol swab and allowed to dry. In the postpartum period only (Visits 3, 4, and 5), maternal body composition will be assessed via bioelectrical impedance analysis (Omron HBF-306C) to estimate fat mass and body fat percentage; this will be performed in only those participants without metal implants or pacemakers.

For infants, anthropometric measurements will be collected during Visits 2–5. Infant length and weight will be assessed with a length board and digital scale (SECA, Hamburg, Germany), with infants weighed in clean diapers after zeroing the scale for diaper weight. Head circumference will be measured at the widest possible point, and abdominal circumference will be measured above the belly button to avoid the umbilical cord stump in early assessments, using a standard tape measure (SECA, Hamburg, Germany). Measurements will be taken in duplicate and averaged for analyses. Age- and sex-standardized BMI z-scores will be calculated based on World Health Organization Child Growth Standards [44,45]. Additionally, abdominal circumference-to-length ratio will be calculated as an indicator of abdominal visceral fat, determined by dividing abdominal circumference by length. Skinfold thicknesses at the tricep, bicep, subscapular, iliac crest, and thigh will be measured on the left side of the body (to minimize potential variability due to limb dominance) using a Harpenden caliper (West Sussex, UK), and subcutaneous fat mass (mm^2^) will be estimated from the sum of all skinfold measurements.

#### Biospecimen and environmental sample collection.

**Saliva:** Saliva specimens will be collected from both mothers and infants using Oragene saliva collection kits for DNA and RNA stabilization (DNA Genotek). Mothers will provide saliva by spitting directly into the tube (DNA, OGR-600) or swabbing their mouth using a kit-supplied absorbent sponge (RNA, ORE-100) under the supervision of trained study staff. Infant saliva specimens will be collected by trained study staff using a kit-supplied absorbent sponge (DNA, OC-175; RNA ORE-100). Once collected, the samples will be mixed with the stabilizing reagent within the tubes to ensure DNA and RNA integrity. Specimens will be securely stored at room temperature until they are shipped in batches to the University of Pittsburgh for DNA and RNA extraction and purification following manufacturer protocols (DNA Genotek). Extracted DNA from maternal and infant saliva samples will be analyzed for *CREBRF* rs373863828 and *BTNL9* rs200884524 using established TaqMan® assays (Applied Biosystems).

**Umbilical cord blood:** Umbilical cord blood specimens will be collected immediately following delivery by trained hospital staff at LBJ Hospital. After clamping and cleaning the umbilical cord, approximately 6 mL of blood will be drawn from the umbilical vein using an 18-gauge needle and sterile syringe. The specimen will then be aseptically transferred into PAXgene Blood DNA tube (Fisher Scientific, Catalog B761165, 2.5 mL) and a PAXgene Blood RNA tube (Fisher Scientific, Catalog 2302101, 2.5 mL). Samples will be frozen at −20°C for 24 hours and then transferred to −80°C storage for long-term preservation. Specimens will be shipped in batches on dry ice to the University of Pittsburgh for DNA and RNA extraction, analysis, and storage. Extracted DNA from cord blood will undergo epigenome-wide DNA methylation analysis using the EPIC v2.0 chip (Illumina).

**Secondary samples:** Several secondary samples will be collected by trained study staff and banked for future research. Capillary blood samples will be obtained from mothers (finger stick) and infants (heel stick) using sterile lancets and Mitra devices (Trajan Scientific Americas Inc.) following manufacturer protocols, with a total volume of up to 120 microliters per sample. The filled devices will be stored at −80°C and batch-shipped to the University of Pittsburgh for future per- and polyfluoroalkyl substance profiling. Infant fecal specimens will be collected using OMNIgene-gut kits (OMR-200, DNA Genotek) following manufacturer protocols, with stool obtained from used diapers before being stored at −80°C for future microbiome profiling. Finally, home water samples will be collected from a household tap (with the tap room location [e.g., kitchen, bathroom] recorded) using pre-cleaned 250 mL polypropylene bottles. Study staff collecting samples will be instructed to avoid using hand lotion on the day of collection and to wear clean nitrile gloves to reduce contamination risk. To monitor for field contamination, blank control samples pre-filled with deionized water will be opened and re-capped at select homes during sampling. All samples will be sealed, placed on ice, and frozen at −20°C within 24 hours and stored for future per- and polyfluoroalkyl substance profiling. All samples will be de-identified to protect participant confidentiality.

#### Study questionnaires.

Participants will complete a series of questionnaires (available in both English and Samoan, side-by-side) that gather information across several key domains including demographic, health, social, behavioral, and environmental factors. Demographic and household questions include topics such as education, relationship status, income, and other family and living characteristics. Health-related questions focus on pregnancy history, pre-pregnancy health, and pregnancy-related conditions, addressing both physical and psychosocial aspects. Behavioral and lifestyle questionnaires assess social support, tobacco and alcohol use, physical activity, nutrition, and sleep health. Environmental questions explore household and community context. Infant-focused sections capture information on feeding practices, sleep patterns, and developmental milestones. Participants will also be invited to share feedback on their study experience and indicate whether they are interested in receiving their genetic results. Additional details about the questionnaires are provided in Table 2. All questionnaire data will be collected using REDCap, a secure, web-based platform hosted on password-protected institutional servers [65,66]. Data will be de-identified to protect participant confidentiality.

**Table 2:** Questionnaires.

Domain	Data Collected	Measure	Visit
Demographic and Background	Maternal age, relationship status, education, household income, occupation	Investigator developed	V1
Pregnancy History and Health	Gestation and due date, pregnancy history, prenatal care, health during pregnancy	Investigator developed	V1
Pre-pregnancy Health	Health history, menstrual cycle information, ocular health	Investigator developed	V1 & V5
Physical and psychosocial health	Physical health, oral health and habits, anxiety, depression, stress, mindfulness, social support, social conflict, perceived discrimination	Investigator developed, Short Form-8 (SF-8) [46,47], Generalized Anxiety Disorder 7-item (GAD-7) [48], Patient Health Questionnaire 9 (PHQ-9) [49,50], Cohen’s Perceived Stress Scale (PSS) [51], Mindful Attention Awareness Scale (MAAS) [52], Multidimensional Scale of Perceived Social Support (MSPSS) [53], Perceived Social Conflict Scale (PSC) [54], Everyday Discrimination Scale (EDS) [48]	V1 & V5
Behavior	Tobacco and alcohol consumption, physical activity, sleep habits, dietary intake	Investigator developed, Pregnancy Physical Activity Questionnaire (PPAQ) [55,56], International Physical Activity Questionnaire (IPAQ) [57], Pittsburgh Sleep Quality Index (PSQI) [58], Epworth Daytime Sleepiness Scale (ESS) [59], Short Healthy Eating Index (sHEI) [58]	V1 & V5
Environment	Home environment (e.g., water quality and access, use of non-stick cookware, fast food consumption), food and resource security	Investigator developed, Household Food Insecurity Access Scale (HFIAS) [60]	V1
Infant birth, feeding, sleeping, milestones	Feeding plan, birth information, infant nutrition, eating behaviors, and sleep habits (e.g., bedtime routine, naps)	Investigator developed, Baby Eating and Behavior Questionnaire (BEBQ) [61], Brief Infant Sleep Questionnaire (BISQ-R SF) [62–64], Ages and Stages Questionnaires (ASQ-3) [61]	V2, V3, V4, & V5

V1 = Visit 1 (Before birth); V2 = Visit 2 (After birth); V3 = Visit 3 (2 months); V4 = Visit 4 (4 months); V5 = Visit 5 (6 months).

#### Medical record review and extraction.

Clinical data will be extracted from participants’ medical records to supplement questionnaire responses and provide additional context. Extracted information will include GDM status, laboratory results (e.g., lipid levels), medication use, anthropometric measurements, birth-related details, and infant growth. Only personnel who are authorized, trained in privacy and data security (including the Health Insurance Portability and Accountability Act), and approved by LBJ Hospital will be permitted to access and extract this information.

#### Participant feedback and referrals to clinical services.

Study-related measurements will not be added to the participants’ medical records. However, participants may choose to receive a personalized “results booklet,” which will be maintained and updated throughout the study for those who opt in. This booklet will include maternal physical measurements (e.g., weight, height, heart rate, blood pressure, and HbA1c) and infant measurements (e.g., weight, length, head circumference, and abdominal circumference) and growth curves (using WHO growth charts). Before providing this information, research assistants will emphasize that all measurements are being collected for research purposes only and do not replace standard clinical care.

For unexpected health findings, participants will be referred to local clinicians at LBJ Hospital. Referral criteria include HbA1c levels ≥6.5%, blood pressure ≥140/90 mmHg (local referral threshold), and severe depression symptoms (Patient Health Questionnaire-9 (PHQ-9) score ≥20 or thoughts of self-harm). In cases of severe hypertension (≥160/110 mmHg) during pregnancy, an urgent preeclampsia evaluation referral will be provided.

Upon study completion, participants may request their own and/or their infant’s *CREBRF* rs373863828 and/or *BTNL9* rs200884524 genotype results. If requested, study staff will reinforce that these results are for research purposes only, may not be fully accurate, and cannot replace certified clinical testing. Participants will also be informed that the genetic data are still being studied and may not be directly useful at this time. The study team will reinforce that recommendations for maintaining a healthy lifestyle remain the same regardless of genotype. Participants will also be encouraged to contact the study team in the future if they have additional questions or would like to be connected with further counseling about their results.

### Data management and statistical analysis

#### Data management, cleaning, and quality control.

All study data will undergo rigorous quality control procedures to ensure accuracy, consistency, and reliability. During data collection, quality assurance will include weekly to monthly reports, data capture review, and routine checks of data entry and protocol adherence. Automated validation rules and branching logic will be used within REDCap to help minimize entry errors in real-time. All modifications to study records will be documented through REDCap’s audit trail functionality. Data management includes secure storage on encrypted, password-protected servers, version-controlled datasets, and restricted access to identifiable information.

All statistical analyses will be conducted in R [67]; examples of specific packages are given below but are subject to change as additional packages are developed. During the analysis phase, data quality diagnostics will be conducted to identify outliers, assess variable distributions, evaluate associations among variables, and examine missing data patterns. If needed, multiple imputation and sensitivity analyses will be employed to assess the impact of missingness.

For omics data, potential biases will be addressed through standardized laboratory controls, including the use of technical replicates, batch-specific standards, and internal efficiency measures. DNA methylation data will be cleaned and quality controlled using our established workflows [68,69] which currently includes application of minfi, lumi, Enmix, funNorm, and ewastools R packages [70–75], though these may be updated as new tools become available. This preprocessing will include batch correction and other normalization techniques to reduce technical variability and increase rigor.

#### Primary and secondary outcomes and data analysis overview.

Aim-specific primary outcomes of the HOPE study include: (1) maternal GDM status, (2) infant anthropometric and body composition measures (length, weight, age- and sex-standardized BMI z-score, head circumference, abdominal circumference, abdominal circumference-to-length ratio, and fat mass estimated from skinfold thicknesses), and (3) infant cord blood DNA methylation. Secondary maternal outcomes include BMI, heart rate, blood pressure, and HbA1c as well as postpartum fat mass estimated via bioelectrical impedance analysis. Across aims, the primary predictor/exposure of interest is maternal-infant *CREBRF* rs373863828 genotype combination. However, analyses will also examine maternal and infant genotypes following an iterative modeling strategy (e.g., modeling outcomes separately for maternal genotype and infant genotype, followed by joint models including both, and then models including an interaction term to test for synergistic effects). Based on the expected genotype distributions, maternal and infant genotypes will be operationalized based on the presence or absence of the minor (A) allele (e.g., AA/AG vs. GG). Linear regression models will be used for continuous outcomes (e.g., anthropometric measures), while binary logistic regression will be used for binary outcomes (e.g., GDM status), adjusting for relevant covariates (detailed below). Multiple testing correction will be made based on the correlation structure of the data using the meff function from the poolr R package [76].

For DNA methylation analyses, epigenome-wide association studies (EWAS) will be conducted to assess associations between DNA methylation with maternal-infant genotype combinations (primary, modeled as described above) and participant characteristics (secondary) using the limma R package [77]. M values (logit-transformed beta values) will be used in linear regression models to identify site-specific associations, adjusting for appropriate covariates. Analyses will be performed both with and without adjustment for cell type heterogeneity [78,79] inferred using established deconvolution methods [80,81]. Multiple testing correction will be applied using a methylome-wide significance threshold (p < 9 × 10⁻⁸). Differentially methylated regions (DMRs) will be identified using EWAS summary statistics and the dmrff R package [82], and gene set enrichment analysis (GSEA), Gene Ontology (GO) enrichment analysis, and Kyoto Encyclopedia of Genes and Genomes (KEGG) pathway analysis will be performed using the missMethyl R package [83,84].

For analyses focused on maternal secondary outcomes or infant outcomes, GDM status will be included as a covariate, with subgroup analyses explored to assess differential effects. Additional covariates (e.g., infant sex, maternal age, gestational age, and social drivers of health) will be included in regression models as appropriate. Additional exploratory analyses are planned to support secondary study goals and future sample collections.

#### Power.

The study sample of up to 180 maternal-infant dyads (accounting for anticipated attrition to achieve a final analytic sample of approximately n = 150 dyads) provides adequate power for the planned analyses. With n = 150 dyads, we have ≥80% power to detect a moderate odds ratio (OR) of 1.75 for the association between maternal-offspring genotype combination and GDM, and a small to moderate effect size (Cohen’s d = 0.28) for associations with continuous infant anthropometric measures. Importantly, these detectable effect sizes are consistent with or smaller than those reported in prior studies of *CREBRF* and metabolic traits in Pacific Islander populations [8,12,14,15]. Power calculations for DNA methylation analyses indicate sufficient power to detect moderate differences at individual CpG sites, even at genome-wide significance thresholds (e.g., ~ 3% difference at up to 25% of sites and ~7% difference at up to 75% of sites), providing critical foundational data for future studies.

### Study status and timeline

Participant recruitment for HOPE began in April 2025. Recruitment is expected to be completed within 12–18 months, with follow-up visits anticipated to conclude within 18–24 months.

## Discussion

This study will support a hypothesis-driven examination of how maternal and infant genetics—particularly the *CREBRF* rs373863828 variant—relate to maternal and infant health in American Samoa. This work is significant because, unlike most genetic variants which have small effect sizes and are often overshadowed by social and behavioral factors, the *CREBRF* variant has demonstrated large and consistent effects [7,8,12,14,85], suggesting potential as a future screening or intervention target. As such, this study may help identify biological factors that contribute to health outcomes among the Pacific Islander population group which may inform population-specific strategies to improve outcomes. Additionally, the study is designed to contribute novel epigenetic and other omic data, offering valuable insight into potential mechanisms linking maternal and infant factors to health outcomes. Importantly, while this variant is enriched in Pacific Islander populations, its involvement in key metabolic pathways suggests broader relevance for understanding metabolic health in diverse populations.

Despite its significance, this study has some limitations related to feasibility, timeline, and resource constraints. For example, recruitment is limited to the third trimester of pregnancy, restricting our ability to examine early pregnancy or pre-conception factors. Similarly, paternal data will not be collected, preventing evaluation of extended family health behaviors or factors that may influence outcomes. In addition, while the use of non-invasive biospecimens was designed to reduce participant burden and distress and increase compliance, it may constrain the types of biomarkers that can be assessed. Finally, the study follows infants only through six months of age, limiting the ability to assess longer-term developmental trajectories.

Despite these constraints, several key strengths enhance the rigor and potential impact of this study. The focus of this work on a historically excluded population group with a high rate of GDM will ensure that findings contribute to reducing health disparities and improving the representation of Pacific Islander communities in genetic and environmental health research. In addition, the longitudinal design supports a comprehensive assessment of factors that shape early infant growth. Finally, the multimodal data collection approach including questionnaires, biospecimens, and genetic and epigenetic analyses enhances the study’s ability to investigate multiple biological pathways influencing maternal and infant health.

Therefore, this study represents a critical first step toward a biologically and culturally grounded understanding of maternal/child health outcomes in American Samoa. It lays the groundwork for future studies that may include earlier pregnancy recruitment, more detailed assessments of environmental and behavioral exposures, and extended follow-up into childhood. Taken together, this line of research may help guide the development of targeted, community-informed interventions to promote maternal and child health in American Samoa and other underrepresented populations globally.
